# Submicroscopic malaria infection during pregnancy and the impact of intermittent preventive treatment

**DOI:** 10.1186/1475-2875-13-274

**Published:** 2014-07-15

**Authors:** Lauren M Cohee, Linda Kalilani-Phiri, Sarah Boudova, Sudhaunshu Joshi, Rabia Mukadam, Karl B Seydel, Patricia Mawindo, Phillip Thesing, Steve Kamiza, Kingsley Makwakwa, Atis Muehlenbachs, Terrie E Taylor, Miriam K Laufer

**Affiliations:** 1 Center for Vaccine Development, School of Medicine, University of Maryland, Baltimore, Maryland, USA; 2 University of Malawi College of Medicine, Blantyre, Malawi; 3 Blantyre Malaria Project, University of Malawi College of Medicine, Blantyre, Malawi; 4 Department of Osteopathic Medical Specialties, College of Osteopathic Medicine, Michigan State University, East Lansing, Michigan, USA; 5 Department of Histopathology, University of Malawi College of Medicine, Blantyre, Malawi; 6 Department of Pathology, University of Washington, Seattle, Washington, USA

**Keywords:** Malaria in pregnancy, Submicroscopic infection, Placental malaria, Sulphadoxine-pyrimethamine intermittent preventive treatment, Sulphadoxine-pyrimethamine resistance

## Abstract

**Background:**

Malaria during pregnancy results in adverse outcomes for mothers and infants. Intermittent preventive treatment (IPT) with sulphadoxine-pyrimethamine (SP) is the primary intervention aimed at reducing malaria infection during pregnancy. Although submicroscopic infection is common during pregnancy and at delivery, its impact throughout pregnancy on the development of placental malaria and adverse pregnancy outcomes has not been clearly established.

**Methods:**

Quantitative PCR was used to detect submicroscopic infections in pregnant women enrolled in an observational study in Blantyre, Malawi to determine their effect on maternal, foetal and placental outcomes. The ability of SP to treat and prevent submicroscopic infections was also assessed.

**Results:**

2,681 samples from 448 women were analysed and 95 submicroscopic infections were detected in 68 women, a rate of 0.6 episodes per person-year of follow-up. Submicroscopic infections were most often detected at enrolment. The majority of women with submicroscopic infections did not have a microscopically detectable infection detected during pregnancy. Submicroscopic infection was associated with placental malaria even after controlling for microscopically detectable infection and was associated with decreased maternal haemoglobin at the time of detection. However, submicroscopic infection was not associated with adverse maternal or foetal outcomes at delivery. One-third of women with evidence of placental malaria did not have documented peripheral infection during pregnancy. SP was moderately effective in treating submicroscopic infections, but did not prevent the development of new submicroscopic infections in the month after administration.

**Conclusions:**

Submicroscopic malaria infection is common and occurs early in pregnancy. SP-IPT can clear some submicroscopic infections but does not prevent new infections after administration. To effectively control pregnancy-associated malaria, new interventions are required to target women prior to their first antenatal care visit and to effectively treat and prevent all malaria infections.

## Background

Each year, 25 million pregnant women in sub-Saharan Africa are at risk for malaria infection. Malaria during pregnancy is associated with maternal anaemia and infant low birth weight. Malaria during pregnancy is estimated to result in 100,000 infant deaths annually in Africa [1].

Malaria infections during pregnancy may be identified by microscopic examination of a blood smear or may be submicroscopic, detectable only by more sensitive molecular methods. Studies in a variety of transmission settings have shown that submicroscopic infections can be up to five times more common than microscopic infections during pregnancy [2-7]. While the associations between microscopically detectable malaria during pregnancy and maternal anaemia at delivery and low birth weight have been supported in most studies [1], the impact of submicroscopic infection during pregnancy on these adverse outcomes has not been systematically assessed.

Because malaria infections during pregnancy are often asymptomatic, a key intervention to decrease the burden of malaria in pregnancy is intermittent preventive treatment (IPT), providing anti-malarial chemotherapy at routine intervals during pregnancy. Currently, the recommended anti-malarial chemotherapy for IPT in the majority of Africa is sulphadoxine-pyrimethamine (SP), which is intended to cure current infections and to provide a period of post-treatment prophylaxis to prevent future infections.

In much of sub-Saharan Africa, increasing resistance to SP [8] is generating concerns that SP-IPT efficacy will be compromised [9]. Understanding the relative importance of the treatment and prophylactic effects of SP-IPT and the differential impact of resistance on these effects may have implications for the selection of a new drug to replace SP for IPT.

In the context of an observational study of a large cohort of pregnant women in Malawi, the effect of submicroscopic infections on maternal and foetal outcomes was examined. All women received SP-IPT according to the national policy. At each visit a blood smear was examined and women were treated for malaria infections detected by microscopy. The primary analysis of infections detected solely by microscopy showed an association between infection at enrolment in antenatal care and placental malaria, but only infections detected at the time of delivery were associated with adverse pregnancy outcomes [10]. Because higher rates of submicroscopic infection than microscopically detectable infection were anticipated and submicroscopic infections were not treated, it was hypothesized that submicroscopic infections would be associated with placental malaria, maternal anaemia and infant low birth weight. It was further hypothesized that, due to high rates of SP resistance, SP-IPT would fail to clear and prevent submicroscopic infections.

## Methods

### Study population

Four-hundred and fifty pregnant women were enrolled in an observational cohort study of malaria during pregnancy in Blantyre, Malawi between June 2009 and June 2010. All women were in their first or second pregnancy and were less than or equal to 28 weeks gestational age based on clinical assessment at enrolment. Women were followed monthly during pregnancy and encouraged to come to the clinic if they had intercurrent illness. At each encounter, peripheral blood smears and dried blood spots on filter paper were collected. At delivery, these same specimens and placental blood and tissue samples were collected. After quickening, women received SP-IPT up to three times separated by at least four weeks. Women with malaria detectable by blood smear at routine or sick visits were treated for malaria in accordance with the national guidelines (quinine in the first trimester and artemether-lumefantrine in the second and third trimesters). Details are described by Kalilani-Phiri *et al.*[10].

### Laboratory procedures

#### Microscopy

Peripheral blood slides were Field’s stained and examined using a 100× oil immersion objective to detect and quantify parasitaemia using an estimated white blood cell count of 8,000 per microlitre. A diagnosis of microscopically detectable malaria infection was made when asexual stage malaria parasites were detected on a thick film. A smear was recorded as negative after examining 100 high power fields. Two microscopists read all slides; in cases of disagreement between the readings, a third expert reader adjudicated.

#### Haemoglobin

Haemoglobin measurement was obtained from a finger prick blood samples using HemoCue® AB.

#### Placental biopsies

Placental biopsies were preserved in 10% neutral buffered formalin, embedded in paraffin wax, cut into four micron thick sections, and then stained with haematoxylin and eosin. Slides were examined for presence of malaria parasites and haemozoin pigment.

#### Molecular detection

DNA was extracted from frozen placental samples and dried blood spots of peripheral blood and placental blood. Quantitative real time polymerase chain reaction (qPCR) was used to detect the gene for *Plasmodium falciparum* lactate dehydrogenase. Extraction and qPCR protocols are described on our website [11].

#### Data analysis

Peripheral blood infections were categorized as either microscopic (smear positive, confirmed by qPCR) or submicroscopic (smear negative, but qPCR positive). An episode of submicroscopic infection was defined as a positive qPCR and a negative malaria smear obtained at the same time. Sequential episodes of submicroscopic parasitaemia were only counted once. Gestational age at enrolment was calculated based on the last menstrual period or by the fundal height if the last menstrual period was not known. Gestational age at birth was determined based on the last menstrual period and the Ballard score. Fundal height at enrolment was included in the estimate of gestational age at birth if the last menstrual period and Ballard estimates were more than two weeks’ discrepant. The first trimester was defined as conception through 13 weeks and the second trimester was from 14 through 27 weeks. Low birth weight was defined as birth weight less than 2,500 g. To further investigate low birth weight, preterm birth and small for gestational age were analysed separately. If the gestational age at delivery was less than 37 weeks, the delivery was classified as preterm. Infants were considered small for gestational age if the birth weight for gestational age, based on WHO growth curves, was less than a Z-score of −2. Fever was defined as a measured axillary temperature ≥37.5°C. Maternal anaemia was defined as haemoglobin <11.0 g/dL. Placental malaria was classified as the presence of haemozoin pigment or parasites, by either histology or qPCR. Submicroscopic placental malaria was defined as parasites detected by qPCR, but not histology. Only women with both molecular and histological placental results were included in descriptive analyses of placental malaria. However, women missing histological results were included in analysis of the presence or absence of placental malaria because detection of parasites by qPCR alone is sufficient to categorize them as having placental malaria. Data from twin gestations were included in analyses of placental malaria but excluded from analyses of birth outcomes. Analyses of the effects of SP were restricted to visits that occurred within 28 days after the first visit.

Data analysis was performed using STATA version 12.1 software (Stata Corp, College Station, TX, USA). Student’s t-tests or Wilcoxon rank-sum were used for comparisons of normal and non-normal distributions of continuous variables, respectively. Chi-squared and Fisher’s exact tests were used for comparisons of proportions. Odds ratios were calculated using univariate and multivariate logistic regression. Analyses of haemoglobin during pregnancy were controlled for gestational age and adjusted for repeated measurements using robust cluster variance estimation. All P-values are two-sided, and statistical significance was set at P ≤0.05.

#### Ethical considerations

Ethical approval was obtained from the University of Malawi College of Medicine Research and Ethics Committee and the University of Maryland Baltimore Institutional Review Board. Written informed consent was obtained from all participants before conducting any study related activities. Participants had the option to withdraw from the study at any time. All data were recorded and analysed anonymously.

## Results

Two thousand six hundred and eighty-one peripheral blood samples from 448 women enrolled in the study were screened for submicroscopic infection. Two women were not included in the analysis because they did not have any filter papers available for molecular analysis. Ninety-five incident submicroscopic infections were detected in 68 women. Pregnant women had 0.6 episodes of submicroscopic malaria per person-year of follow-up. As previously published, women in this cohort also experienced 0.6 episodes of microscopically detected infection per person-year of follow-up [10]. Thus, the overall rate of malaria infection was 1.2 episodes per person year of follow up.

**Figure 1:**
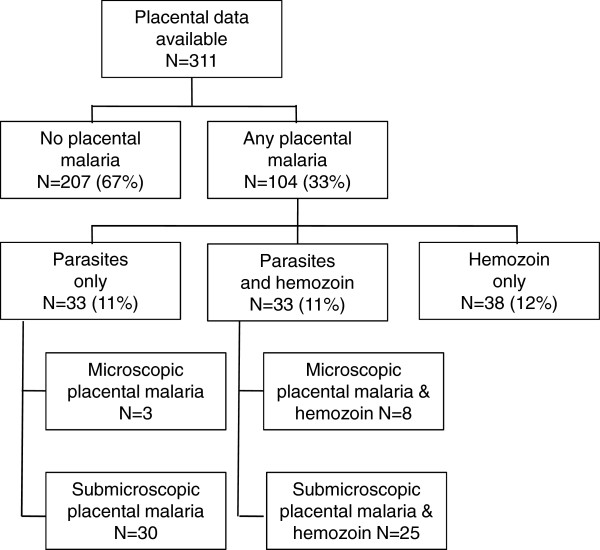
Frequency of histological and molecular findings of placental malaria.

Among women with submicroscopic infections, the mean number of infections was 1.4 (95% CI 1.2-1.6, range 1–5). Forty-nine women (72%) with submicroscopic infections never had an infection detected by microscopy during pregnancy. Submicroscopic infection was not associated with first *versus* second pregnancy, age, bed net use, or malaria treatment during pregnancy prior to enrolment, but was associated with enrolment at an earlier gestational age, more frequent visits, and lack of secondary school attendance (Table 1). Gestational age at enrolment was inversely correlated with number of visits.Among 311 placentas with both histological and molecular data available for evaluation, 104 (33%) had evidence of placental malaria (Figure 1). Thirty-three (11%) had both parasites and haemozoin detected microscopically. Three (1%) had no haemozoin but did have parasites detected by histology and confirmed by qPCR. Thirty-eight (12%) had haemozoin without histological or molecular evidence of active parasite infection. Thirty (10%) had submicroscopic infection with no histological evidence of parasites or haemozoin.

**Table 1:** Enrolment characteristics of women with and without submicroscopic infection detected during pregnancy irrespective of the presence of microscopic infection

	**Submicroscopic infection (N = 68)**	**No submicroscopic infections (N = 380)**	**p-value**
Mean gestational age in weeks (95% CI)	18.9 (17.8-20.1)	20.3 (19.9-20.7)	0.04
Mean age in years (95% CI)	20.1 (19.3-20.8)	20.1 (19.8-20.4)	0.9
Primigravid (%)	37 (54%)	247 (65%)	0.1
Did not attend secondary school (%)	33 (49%)	106 (28%)	0.001
Slept under bed net last night (%)	31 (46%)	194 (51%)	0.3m
Treated for malaria during pregnancy but prior to enrolment (%)	8 (12%)	57 (15%)	0.4
Mean number of visits (95% CI)	6.4 (5.6-7.2)	5.4(5.1-5.7)	0.01

CI: confidence interval.

Histological results were not available for six placentas; all had parasites detected by qPCR.

### Timing and clinical presentation of submicroscopic infection

Among women with at least one submicroscopic infection, 69% had submicroscopic infection detected at enrolment (47/68). Submicroscopic infections were detected in each trimester and at delivery. Based on estimated gestational age at enrolment, submicroscopic infection was detected in 12% (6/50) of first trimester, 6% (63/1,050) of second trimester, and 1.4% (19/1,372) of third trimester samples prior to delivery. These infections occurred in six, fifty-six, and eighteen women, respectively. Twelve women had infection in more than one trimester. After excluding the submicroscopic infections present at enrolment, the prevalence of submicroscopic infection was higher in the second trimester than the third trimester [22/650 (3.4%) *versus* 19/1,372 (1.4%) of samples, p = 0.003].

Nine of 308 women (3%) with peripheral samples obtained at delivery had submicroscopic infection. Two women with submicroscopic infection at delivery also had submicroscopic infection at their last antepartum visit, thus infection at delivery was not considered a new submicroscopic infection.

Submicroscopic infection was not associated with documented fever (p = 0.4) or reported fever within 48 hours prior to the visit (p = 0.2). Three women reported cough, sneezing and body pains at a visit during which submicroscopic infection was detected. Otherwise, no women reported any symptoms of illness during visits at which submicroscopic infection was detected. However, submicroscopic infection was associated with a 0.6 g/dL decrease in mean haemoglobin at the time of detection (11.5 g/dL *vs* 12.1 g/dL, p = 0.001). This decrease remained significant after adjusting for gestational age and repeated measurements.

### Impact of submicroscopic infection

Women who had submicroscopic infections were more likely to have placental malaria than those without infections. After controlling for microscopically detectable infection, women with submicroscopic infection were more than seven-fold more likely to have placental malaria than women without any documented infection during pregnancy (Table 2). Each additional episode of submicroscopic infection increased the risk of placental malaria nearly five-fold (OR 4.9, 95% CI 2.7-9.1, p < 0.001). All women with submicroscopic infection detected in peripheral blood at delivery had placental malaria. Thirty-five percent (39/110) of women who had evidence of placental malaria did not have any microscopic or submicroscopic peripheral infections detected during pregnancy or at delivery.

**Table 2:** Association of submicroscopic infection during pregnancy and placental malaria

	**Any placental malaria**	**No placental malaria**	**Odds ratio (95% CI)**
No infection	39/110 (35%)	184/207 (89%)	Referent
Submicroscopic infection	37/110 (34%)	14/207 (7%)	7.5 (3.6-15.6)
Microscopic infection	49/110 (45%)	10/207 (5%)	16.6 (7.7-35.8)

CI: confidence interval.

Among women without microscopic infection during pregnancy, submicroscopic infection detected at enrolment was more strongly associated with placental malaria than submicroscopic infection detected after enrolment. After excluding infections detected at enrolment, third trimester infections were more strongly associated with placental malaria than second trimester infections (Table 3).

**Table 3:** Timing of submicroscopic infection during pregnancy and odds of placental malaria among women without microscopically detected infection

**Timing of submicroscopic infection detection**	**Any placental malaria**	**No placental malaria**	**Odds ratio (95% CI)**
No submicroscopic infection	39/61 (64%)	184/197 (93%)	Referent
Submicroscopic infection at enrolment visit*	19/60 (32%)	9/194 (5%)	6.9 (2.7-17)
Submicroscopic infection at a follow-up visit in the 2^nd^ trimester	6/61 (10%)	3/197 (2%)	1.9 (0.3-12)
Submicroscopic infection at a follow-up visit in the 3^rd^ trimester	7/61 (11%)	2/197 (1%)	3.5 (0.5-23)

*Four women did not have molecular data available from their enrolment visit.

CI: confidence interval.

Submicroscopic infection during pregnancy, at delivery or in the placenta was not associated with adverse maternal or foetal outcomes. The overall rates of these adverse outcomes were: 18% low birth weight, 13% small for gestational age, 15% preterm delivery and 11% maternal anaemia at delivery. The prevalence of these outcomes was the same in women who did and did not have submicroscopic infection in their peripheral blood or placenta (Table 4).

**Table 4:** Maternal and foetal outcomes in singleton, live births from women with only submicroscopic infection compared to women without infection during pregnancy, at delivery or in the placenta

	**Only submicroscopic infection during pregnancy (N = 35)**	**No infection during pregnancy (N = 228)**	**p-value**	**Submicroscopic infection at delivery (N = 7)**	**No infection at delivery (N = 267)**	**p-value**	**Submicroscopic placental malaria (N = 52)**	**No placental malaria (N = 198)**	**p-value**
Mean birth weight in kg (95% CI)	2.9 (2.7-3.1)	2.8 (2.78-2.90)	0.7	3.2 (2.9-3.6)	2.8 (2.8-2.9)	0.3	2.8 (2.6-2.9)	2.8 (2.8-2.9)	0.5
Low birth weight (%)	5 (14%)	40 (17%)	0.6	1 (11%)	42 (16%)	1.0	12 (23%)	31 (16%)	0.3
Mean gestational age in weeks (95% CI)	39 (37–40)	39 (38-39)	1.0	40 (39-41)	39 (38-39)	0.3	39 (38-40)	39 (38-39)	0.8
Preterm delivery (%)	2 (6%)	40 (17%)	0.1	0 (0%)	35 (13%)	0.6	6 (12%)	25 (13%)	0.8
Small for gestational age (%)	2 (6%)	29 (13%)	0.4	0 (0%)	28 (10%)	1.0	8 (15%)	20 (10%)	0.3
Mean maternal [Hb] in g/dL at delivery (95% CI)	12.9 (12.4-13.3)	12.9 (12.7-13.1)	1.0	12.7 (11.0-14.3)	12.9 (12.8-13.1)	0.6	12.9 (12.5-13.3)	12.9 (12.7-13.1)	0.9
Maternal anemia at delivery (%)	3 (9%)	21 (9%)	1.0	1 (14%)	23 (9%)	0.5	3 (6%)	16 (8%)	0.8

CI: confidence interval.

[Hb]: haemoglobin concentration.

### Effect of SP-IPT on submicroscopic infections

The ability of SP to clear submicroscopic infections was evaluated by examining women who had submicroscopic infection and a subsequent visit within 28 days (N = 79 visits). Women who received a dose of SP when they had a submicroscopic infection were less likely to have an infection at their next visit within 28 days than women with submicroscopic infections who did not receive SP (23.9 *vs* 48.5%; p = 0.02).

**Figure 2:**
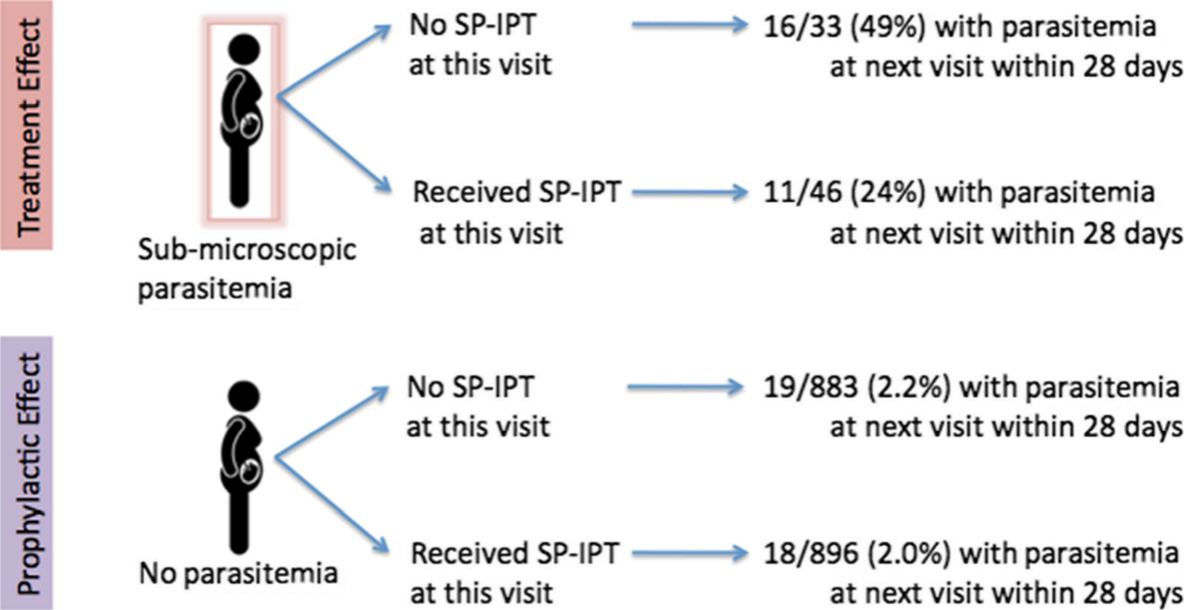
SP-IPT treatment of submicroscopic infection and prevention of any infection.

The prophylactic efficacy of SP was assessed by comparing the rates of malaria infection within 28 days in women who did and did not receive SP when they had no malaria infection. A total of 1,779 visits were included in the analysis. Women who did and did not receive SP had the same rate of parasitaemia within the next 28 days (2.0 *vs* 2.2%; p = 0.83) (Figure 2).

## Discussion

Even with adherence to SP-IPT administration three times during pregnancy and active case detection and treatment of malaria, submicroscopic infection of the peripheral blood during pregnancy is frequent, especially at the first antenatal visit. Low-density malaria infections, detected only by molecular methods, are associated with placental malaria infection and a decrease in maternal haemoglobin levels. SP-IPT appears to treat some submicroscopic infections but does not prevent new infections. These findings have important implications for the development of further interventions for prevention of malaria pregnancy.

This is the first report of the effect of submicroscopic infection throughout pregnancy on maternal and foetal outcomes. Prior studies of submicroscopic malaria during pregnancy have been limited by being cross-sectional, rather than longitudinal in nature [2,3,6,7,12-15]. Linking antenatal infections to specific delivery and neonatal outcomes is essential to evaluating the importance of submicroscopic infections and designing strategies to optimize maternal and infant health.

The results are consistent with previous studies indicating that submicroscopic infection is associated with anaemia at the time of detection [12,15]. Two previous studies reported an association between submicroscopic infection at delivery and infant low birth weight [6] or maternal anaemia at the time of delivery [7]. However, these cross-sectional studies took place prior to, or as part of, IPT trials and did not assess previous exposure to or treatment of malaria infection during pregnancy. Thus, submicroscopic infection may have been a marker of frequent malaria exposure or lack of IPT administration prior to delivery. Due to detailed follow-up and treatment of microscopically detectable infection, the results of this study more accurately reflect the specific contribution of submicroscopic infections to clinical outcomes.

The lack of association between submicroscopic infection during pregnancy and adverse outcomes despite the strong association with placental malaria may have been a function of the design of the study and the clinical care the participants received. The results from the examination of the effect of microscopically detectable infections on maternal and foetal outcomes yielded similar results, as published previously [10]. The combination of three doses of SP-IPT and active case detection and treatment may have protected against adverse effects. It is also possible that other interventions, such as screening for anaemia or the appropriate treatment of non-malaria infections, might have improved the overall health of women, decreasing the frequency of adverse outcomes. Given the low prevalence of adverse outcomes, sample size also limited the ability to detect associations. Previous studies have demonstrated a 50% increase in rates of low birth weight [6] and maternal anaemia [7] at delivery associated with malaria during pregnancy. This study had only 15 and 7% power to detect a 50% increase in low birth weight and maternal anaemia at delivery, respectively, given the baseline rates of these outcomes in this cohort.

The presence of placental malaria infection in women who did not have any peripheral malaria infection detected during pregnancy was unexpected. This phenomenon may be due to transient infections that were not captured during sampling intervals. Results from this study indicate that pregnant women frequently clear submicroscopic infections spontaneously. Another source of the placental malaria infection may be infection that occurred prior to enrolment in antenatal care. The strong association between malaria at enrolment and placental infection suggests that early infection, even those that had been cleared at the time of the first antenatal visit, may have the greatest likelihood of causing placental pathology.

The modest efficacy of SP in clearing submicroscopic infection and the lack of prophylactic efficacy likely reflects the high level of SP resistance among malaria parasites in Malawi [8,16]. This is consistent with studies of SP-IPT in infants, that showed the protective effect of SP was approximately one month and the duration of this protective efficacy is inversely related to presence of *in vitro* markers of SP resistance [17,18].

There are several important features of this study that may limit the generalizability of the findings. Women had an average of six visits during the study and were screened with microscopy at each encounter. Thus, women received more screening and treatment when participating in the study than they would have during routine antenatal care. Despite the intensive follow-up and medical attention provided in the study, submicroscopic infection is strongly associated with placental malaria and anaemia during pregnancy. In real life settings, the association between submicroscopic malaria and adverse outcomes would likely have been more pronounced. Having few encounters during the first trimester and having samples obtained intermittently limited analysis of the timing of infection leading to placental infection. Women with submicroscopic infection did have more visits during the study, suggesting that increasing sampling frequency would increase detection of submicroscopic infection. Alternatively, women with submicroscopic infections may have symptoms leading them to present to the antenatal clinic more frequently. However, there was not an association between submicroscopic infection and fever or routine *versus* sick visit.

These results have important implications for the development of alternatives to SP-IPT for prevention of malaria pregnancy. Two possible strategies are intermittent screening and treatment or IPT with an anti-malarial more effective than SP. In one trial, intermittent screening and treatment has been shown to be equivalent to SP-IPT [19]. The intermittent screening and treatment arm in that study used antigen-detecting rapid diagnostic tests for screening. The parasite density threshold for detection for rapid diagnostic tests, like that for microscopy, is ten- to 100-fold higher than for qPCR [20,21]. Rapid diagnostic tests, like microscopy, will fail to detect infection at the submicroscopic level. Thus, a rapid diagnostic test-based screening and treatment strategy will fail to detect infections that this study demonstrates are associated with placental malaria. Within the context of the usual antenatal package of four antenatal visits in pregnancy if IPT is not provided, many microscopic and all submicroscopic infections will not be treated. Under these conditions, with prolonged and untreated episodes of low-density malaria infection, it is expected that the adverse effects of placental malaria would occur [22-24].

If IPT with a more effective anti-malarial is considered, for example as in ongoing trials of IPT with dihydroartemisinin-piperaquine [25], azithromycin combined with chloroquine [26] and mefloquine [27], it will be important to characterize the treatment and prophylactic effect of candidate drugs. Most drug efficacy trials examine the ability of a drug to cure the infection at the time of drug administration. However, IPT is most beneficial in high transmission settings if it cures current infections and prevents subsequent infections. The results of this study indicate that the loss of prophylactic efficacy may precede the loss of curative efficacy. Thus, in trials of alternative anti-malarials for IPT, prophylactic efficacy should be included as an outcome measure.

The finding that the highest rates of asymptomatic and submicroscopic malaria are detected in women at their first antenatal care visit highlights an essential shortcoming of the current policies to prevent pregnancy-associated malaria. Interventions such as the distribution of bed nets at antenatal clinics and the administration of IPT are delayed until women seek antenatal care. In Malawi, as in other countries in sub-Saharan Africa, this is typically late in the second or into the third trimester [28]. By the time women have their first antenatal visit, peripheral and placental infection has already been established. Innovative interventions that can target women early in pregnancy, or even prior to pregnancy, are urgently needed to further decrease the burden of malaria during pregnancy.

## Conclusions

Submicroscopic malaria infection is common, occurs early in pregnancy and is associated with placental malaria. SP-IPT appears to treat some submicroscopic infections but does not prevent new infections. To effectively control pregnancy-associated malaria, new interventions are required to target women prior to their first antenatal care visit and to effectively treat and prevent all malaria infections.

## Abbreviations

IPT: Intermittent preventive treatment; SP: Sulphadoxine-pyrimethamine; SP-IPT: Intermittent preventive treatment with sulphadoxine-pyrimethamine; PCR: Polymerase chain reaction; qPCR: Quantitative polymerase chain reaction.

## Competing interests

The authors have declared that they have no financial disclosures or competing interests.

## Authors’ contributions

LKP, TET and MKL conceived of and designed the field study. LKP, TET, MKL, PCT, MM, PW, and GM conducted the clinical study. LMC and MKL designed the experiments. KM, SK and AM completed the histopathology. LMC, SB, RM, SJ, and KBS conducted the experiments. LMC and MKL performed the analysis and led the writing of the manuscript. All authors read and approved the final manuscript.

## References

[B1] Desai M ter Kuile FO Nosten F McGready R Asamoa K Brabin B Newman RD Epidemiology and burden of malaria in pregnancy Lancet Infect Dis 2007 7 93 104 17251080 10.1016/S1473-3099(07)70021-X

[B2] Rantala A-M Taylor SM Trottman PA Luntamo M Mbewe B Maleta K Kulmala T Ashorn P Meshnick SR Comparison of real-time PCR and microscopy for malaria parasite detection in Malawian pregnant women Malar J 2010 9 269 20925928 10.1186/1475-2875-9-269 PMC2984567

[B3] Walker-Abbey A Djokam RRT Eno A Leke RFG Titanji VPK Fogako J Sama G Thuita LH Beadslee E Snounou G Zhou A Taylor DW Malaria in pregnant Cameroonian women: the effect of age and gravidity on submicroscopic and mixed-species infections and multiple parasite genotypes Am J Trop Med Hyg 2005 72 229 235 15772312

[B4] Mayengue PI Rieth H Khattab A Issifou S Kremsner PG Klinkert M-Q Ntoumi F Submicroscopic *Plasmodium falciparum* infections and multiplicity of infection in matched peripheral, placental and umbilical cord blood samples from Gabonese women Trop Med Int Health 2004 9 949 958 15361107 10.1111/j.1365-3156.2004.01294.x

[B5] Malhotra I Dent A Mungai P Muchiri E King CL Real-time quantitative PCR for determining the burden of *Plasmodium falciparum* parasites during pregnancy and infancy J Clin Microbiol 2005 43 3630 3635 16081889 10.1128/JCM.43.8.3630-3635.2005 PMC1234007

[B6] Adegnike AA Verweji JJ Agnandji ST Chai SK Breitling LP Ramharter M Frolich M Issifou S Kremsner PG Yazdanbakhsh M Microscopic and sub-microscopic *Plasmodium falciparum* infection, but not inflammation caused by in fection, is associated with low birth weight Am J Trop Med Hyg 2006 75 798 803 17123968

[B7] Mayor A Moro L Aguilar R Bardaji A Cistero P Serra-Casas E Sigauque B Alonso PL Ordi J Menendez C How hidden can malaria be in pregnant women? Diagnosis by microscopy, placental histology, polymerase chain reaction and detection of histidine-rich protein 2 in plasma Clin Infect Dis 2012 54 1561 1568 22447794 10.1093/cid/cis236

[B8] Sridaran S McClintock SK Syphard LM Herman KM Barnwell JW Udhayakumar V Anti-folate drug resistance in Africa: meta-analysis of reported dihydrofolate reductase (dhfr) and dihydropteroate synthase (dhps) mutant genotype frequencies in African *Plasmodium falciparum* parasite populations Malar J 2010 9 247 20799995 10.1186/1475-2875-9-247 PMC2940896

[B9] Harrington W McGready R Muehlenbachs A Fried M Nosten F Duffy P Intermittent preventive treatment in pregnancy with sulfadoxine-pyrimethamine: the times they are a-changin’ Clin Infect Dis 2012 55 1025 1026 author reply 1026–7 22715177 10.1093/cid/cis568

[B10] Kalilani-Phiri L Thesing PC Nyirenda OM Mawindo P Madanitsa M Membe G Wylie B Masonbrink A Makwakwa K Kamiza S Muehlenbachs A Taylor TE Laufer MK Timing of malaria infection during pregnancy has characteristic maternal, infant and placental outcomes PLoS One 2013 8 e74643 24058614 10.1371/journal.pone.0074643 PMC3776740

[B11] University of Maryland Center for Vaccine Development Malaria Group Protocolshttp://medschool.umaryland.edu/malaria/protocols.asp

[B12] Mayor A Serra-Casas E Bardají A Sanz S Puyol L Cisteró P Sigauque B Mandomando I Aponte JJ Alonso PL Menéndez C Sub-microscopic infections and long-term recrudescence of *Plasmodium falciparum* in Mozambican pregnant women Malar J 2009 8 9 19134201 10.1186/1475-2875-8-9 PMC2633011

[B13] Adam I A-Elbasit IE Salih I Elbashir MI Submicroscopic *Plasmodium falciparum* infections during pregnancy, in an area of Sudan with a low intensity of malaria transmission Ann Trop Med Parasitol 2005 99 339 344 15949181 10.1179/136485905X36244

[B14] Saute F Menendez C Mayor A Aponte J Gomez-Olive X Dgedge M Alonso P Malaria in pregnancy in rural Mozambique: the role of parity, submicroscopic and multiple *Plasmodium falciparum* infections Trop Med Int Health 2002 7 19 28 11851951 10.1046/j.1365-3156.2002.00831.x

[B15] Mockenhaupt FP Rong B Till H Eggelte TA Beck S Gyasi-Sarpong C Thompson WNA Bienzle U Submicroscopic *Plasmodium falciparum* infections in pregnancy in Ghana Trop Med Int Health 2000 5 167 173 10747278 10.1046/j.1365-3156.2000.00532.x

[B16] Taylor SM Antonia A Feng G Mwapasa V Chaluluka E Molyneux M ter Kuile FO Rogerson SJ Meshnick SR Adaptive evolution and fixation of drug-resistant *Plasmodium falciparum* genotypes in pregnancy-associated malaria: 9-year results from the QuEERPAM study Infect Genet Evol 2012 12 282 290 22119749 10.1016/j.meegid.2011.11.006 PMC3293939

[B17] Cairns M Gosling R Carneiro I Gesase S Mosha JF Hashim R Kaur H Lemnge M Mosha FW Greenwood B Chandramohan D Duration of protection against clinical malaria provided by three regimens of intermittent preventive treatment in Tanzanian infants PLoS One 2010 5 e9467 20209126 10.1371/journal.pone.0009467 PMC2830887

[B18] Griffin JT Cairns M Ghani AC Roper C Schellenberg D Carneiro I Newman RD Grobusch MP Greenwood B Chandramohan D Gosling RD Protective efficacy of intermittent preventive treatment of malaria in infants (IPTi) using sulfadoxine-pyrimethamine and parasite resistance PLoS One 2010 5 e12618 20838642 10.1371/journal.pone.0012618 PMC2935388

[B19] Tagbor H Bruce J Agbo M Greenwood B Chandramohan D Intermittent screening and treatment versus intermittent preventive treatment of malaria in pregnancy: a randomised controlled non-inferiority trial PLoS One 2010 5 e14425 21203389 10.1371/journal.pone.0014425 PMC3010999

[B20] WHO Results of WHO product testing of malaria RDTs: Round 4 (2012) [http://www.finddiagnostics.org/export/sites/default/resource-centre/reports_brochures/docs/RDTMalariaRd4_Web3.pdf]

[B21] Alemayehu S Feghali KC Cowden J Komisar J Ockenhouse CF Kamau E Comparative evaluation of published real-time PCR assays for the detection of malaria following MIQE guidelines Malar J 2013 12 277 23927553 10.1186/1475-2875-12-277 PMC3750446

[B22] Steketee RW Nahlen BL Parise ME Menendez C The burden of malaria in pregnancy in malaria-endemic areas Am J Trop Med Hyg 2001 64 1–2 Suppl 28 35 11425175 10.4269/ajtmh.2001.64.28

[B23] Guyatt HL Snow RW Impact of malaria during pregnancy on low birth weight in sub-Saharan Africa Clin Microbiol Rev 2004 17 760 769 15489346 10.1128/CMR.17.4.760-769.2004 PMC523568

[B24] Van Geertruyden J-P Thomas F Erhart A D’Alessandro U The contribution of malaria in pregnancy to perinatal mortality Am J Trop Med Hyg 2004 71 2 Suppl 35 40 15331817

[B25] Efficacy of Intermittent Screening and Treatment or Intermittent Preventive Treatment (IPT) With Dihydroartemisinin-Piperaquine, Versus IPT With Sulfadoxine-Pyrimethamine for the Control of Malaria in Pregnancy in Kenya - Full Text View - ClinicalTrials.g [http://clinicaltrials.gov/ct2/show/NCT01669941]

[B26] Chandra RS Orazem J Ubben D Duparc S Robbins J Vandenbroucke P Creative solutions to extraordinary challenges in clinical trials: methodology of a phase III trial of azithromycin and chloroquine fixed-dose combination in pregnant women in Africa Malar J 2013 12 122 23577619 10.1186/1475-2875-12-122 PMC3636121

[B27] Evaluation of Alternative Antimalarial Drugs for Malaria in Pregnancy - Full Text View - ClinicalTrials.gov [http://clinicaltrials.gov/ct2/show/NCT00811421?term=mefloquine+malaria+prevention+pregnancy&rank=1]

[B28] Antenatal care in developing countries: promises, achievements and missed opportunities. An analysis of trends, levels and differentials, 1990–2001 [http://whqlibdoc.who.int/publications/2003/9241590947.pdf]

